# Effect of Dapagliflozin on Outpatient Worsening of Patients With Heart Failure and Reduced Ejection Fraction: A Prespecified Analysis of DAPA-HF

**DOI:** 10.1161/CIRCULATIONAHA.120.047480

**Published:** 2020-09-04

**Authors:** Kieran F. Docherty, Pardeep S. Jhund, Inder Anand, Olof Bengtsson, Michael Böhm, Rudolf A. de Boer, David L. DeMets, Akshay S. Desai, Jaroslaw Drozdz, Jonathan Howlett, Silvio E. Inzucchi, Per Johanson, Tzvetana Katova, Lars Køber, Mikhail N. Kosiborod, Anna Maria Langkilde, Daniel Lindholm, Felipe A. Martinez, Béla Merkely, Jose C. Nicolau, Eileen O’Meara, Piotr Ponikowski, Marc S. Sabatine, Mikaela Sjöstrand, Scott D. Solomon, Sergey Tereshchenko, Subodh Verma, John J.V. McMurray

**Affiliations:** 1 British Heart Foundation Cardiovascular Research Centre, University of Glasgow, UK (K.F.D., P.S.J., J.J.V.M.).; 2 Department of Cardiology, University of Minnesota, Minneapolis (I.A.).; 3 Late Stage Development, Cardiovascular, Renal, and Metabolism, BioPharmaceuticals R&D, AstraZeneca, Gothenburg, Sweden (O.B., P.J., A.M.L., D.L., M.S.).; 4 Department of Medicine, Saarland University Hospital, Homburg/Saar, Germany (M.B.).; 5 Department of Cardiology, University Medical Center Groningen, University of Groningen, The Netherlands (R.A.d.B.).; 6 Department of Biostatistics & Medical Informatics, University of Wisconsin, Madison (D.L.D.).; 7 Cardiovascular Division, Brigham and Women’s Hospital, Boston, MA (A.S.D., S.D.S.).; 8 Department of Cardiology, Medical University of Lodz, Poland (J.D.).; 9 University of Calgary, Cumming School of Medicine and Libin Cardiovascular Institute, Alberta, Canada (J.H.).; 10 Section of Endocrinology, Yale University School of Medicine, New Haven, CT (S.E.I.).; 11 Clinic of Cardiology, National Cardiology Hospital, Sofia, Bulgaria (T.K.).; 12 Department of Cardiology Copenhagen University Hospital, Denmark (L.K.).; 13 Saint Luke’s Mid America Heart Institute and University of Missouri-Kansas City (M.N.K.).; 14 The George Institute for Global Health and University of New South Wales, Sydney, Australia (M.N.K.).; 15 Universidad Nacional de Córdoba, Argentina (F.A.M.).; 16 Heart and Vascular Center, Semmelweis University, Budapest, Hungary (B.M.).; 17 Instituto do Coracao (InCor), Hospital das Clínicas Faculdade de Medicina, Universidade de São Paulo, Brazil (J.C.N.).; 18 Department of Cardiology, Montreal Heart Institute, Ontario, Canada (E.O’M.).; 19 Center for Heart Diseases, University Hospital, Wroclaw Medical University, Poland (P.P.).; 20 TIMI Study Group, Cardiovascular Division, Brigham and Women’s Hospital, Harvard Medical School, Boston, MA (M.S.S.).; 21 Department of Myocardial Disease and Heart Failure, National Medical Research Center of Cardiology, Moscow, Russia (S.T.).; 22 Division of Cardiac Surgery, St. Michael’s Hospital, University of Toronto, Ontario, Canada (S.V.).

**Keywords:** heart failure, hospitalization, sodium-glucose transporter 2 inhibitors, therapy, treatment outcome

## Abstract

In the DAPA-HF trial (Dapagliflozin and Prevention of Adverse Outcomes in Heart Failure), dapagliflozin, added to guideline-recommended therapies, reduced the risk of mortality and heart failure (HF) hospitalization. We examined the frequency and significance of episodes of outpatient HF worsening, requiring the augmentation of oral therapy, and the effects of dapagliflozin on these additional events.

**Methods:**

Patients in New York Heart Association functional class II to IV, with a left ventricular ejection fraction ≤40% and elevation of NT-proBNP (N-terminal pro-B-type natriuretic peptide), were eligible. The primary outcome was the composite of an episode of worsening HF (HF hospitalization or an urgent HF visit requiring intravenous therapy) or cardiovascular death, whichever occurred first. An additional prespecified exploratory outcome was the primary outcome plus worsening HF symptoms/signs leading to the initiation of new, or the augmentation of existing, oral treatment.

**Results:**

Overall, 36% more patients experienced the expanded, in comparison with the primary, composite outcome. In the placebo group, 684 of 2371 (28.8%) patients and, in the dapagliflozin group, 527 of 2373 (22.2%) participants experienced the expanded outcome (hazard ratio, 0.73 [95% CI, 0.65–0.82]; *P*<0.0001). Each component of the composite was reduced significantly by dapagliflozin. Over the median follow-up of 18.2 months, the number of patients needed to treat with dapagliflozin to prevent 1 experiencing an episode of fatal or nonfatal worsening was 16. Among the 4744 randomly assigned patients, the first episode of worsening was outpatient augmentation of treatment in 407 participants (8.6%), an urgent HF visit with intravenous therapy in 20 (0.4%), HF hospitalization in 489 (10.3%), and cardiovascular death in 295 (6.2%). The adjusted risk of death from any cause (in comparison with no event) after an outpatient worsening was hazard ratio, 2.67 (95% CI, 2.03–3.52); after an urgent HF visit, the adjusted risk of death was hazard ratio, 3.00 (95% CI, 1.39–6.48); and after a HF hospitalization, the adjusted risk of death was hazard ratio, 6.21 (95% CI, 5.07–7.62).

**Conclusion:**

In DAPA-HF, outpatient episodes of HF worsening were common, were of prognostic importance, and were reduced by dapagliflozin.

**Registration:**

URL: https://www.clinicaltrials.gov; Unique Identifier: NCT03036124.

## Abstract 2

Supplemental Digital Content is available in the text.

**Clinical Perspective**

**What Is New?**

- In this prespecified analysis of the DAPA-HF trial (Dapagliflozin and Prevention of Adverse Outcomes in Heart Failure), we found that, in comparison with placebo, the sodium-glucose transporter 2 inhibitor dapagliflozin reduced the risk of an episode of outpatient worsening of heart failure (HF) requiring intensification of oral HF therapy by 26%.
- Episodes of outpatient worsening of HF were common, affecting 1 in 8 patients during a median follow-up of 18 months and were prognostically important: after an outpatient intensification of HF therapy, patients had a ≈3-fold higher risk of death from any cause than patients experiencing no nonfatal manifestations of HF during follow-up.

**What Are the Clinical Implications?**

- The frequency of episodes of outpatient HF worsening requiring intensification of oral therapy, along with their prognostic importance and the availability of a therapeutic intervention to reduce them, should highlight their importance to clinicians and encourage preventative action.
- These findings may have potential consequences for the design of clinical trials: current outcome definition guidelines include only outpatient HF worsening events that are treated with intravenous therapy.
- Outpatient worsening events treated with oral therapy are more common than those treated with intravenous therapy and have similar prognostic significance; therefore, they should be considered for inclusion in future clinical trial outcome definitions.

The most commonly used outcome in clinical trials in patients with heart failure and reduced ejection fraction (HFrEF) is the composite of time to hospitalization for worsening heart failure or cardiovascular death, whichever occurs first.^1–4^ In recent years there has been a major effort to reduce the rate of hospital admission by treating worsening heart failure outside the traditional inpatient ward setting. This was recognized in the updated Standardized Data Collection for Cardiovascular Trials Initiative cardiovascular and stroke end point definitions for clinical trials that included episodes of worsening heart failure if they resulted in intravenous treatment.^5–7^ However, such events are infrequent, even in contemporary trials.^8–11^ Many more patients are treated for worsening symptoms and signs in the community by means of the augmentation of oral therapy.^11,12^ The frequency and prognostic importance of such nonhospitalized episodes of worsening, treated with oral therapy, is not clear, in part, because these episodes have not been recorded in many trials and, where they have been recorded, such events have not been sought systematically or defined in a consistent way. ^8–12^ In the placebo-controlled DAPA-HF trial (Dapagliflozin and Prevention of Adverse Outcomes in Heart Failure), dapagliflozin, added to other guideline-recommended therapies, reduced the risk of mortality and heart failure hospitalization, and improved symptoms in 4744 patients with HFrEF.^13–15^ Episodes of outpatient worsening were collected systematically in DAPA-HF, and the analysis of these episodes was prespecified. We report the frequency of episodes of outpatient worsening, the treatment given for them, the prognostic importance of these events, and the effect of dapagliflozin on them.

## Methods

DAPA-HF was a prospective, randomized, double-blind, controlled trial in patients with HFrEF that evaluated the efficacy and safety of dapagliflozin 10 mg once daily, in comparison with matching placebo, added to standard care. The design, baseline characteristics, and primary results of the trial have been published.^13–15^ Ethics Committees at each of the 410 participating institutions (in 20 countries) approved the protocol, and all patients gave written informed consent. Data underlying the findings described in this article may be obtained in accordance with AstraZeneca’s data sharing policy.^16^

### Study Patients

Men and women aged ≥18 years who had heart failure (HF) were eligible if they were in New York Heart Association (NYHA) functional class II to IV, had a left ventricular ejection fraction ≤40%, and were optimally treated with pharmacological and device therapy for HFrEF. Participants were also required to have an NT-proBNP (N-terminal pro-B-type natriuretic peptide) concentration ≥600 pg/mL (≥400 pg/mL if hospitalized for HF within the previous 12 months). Patients with atrial fibrillation or atrial flutter were required to have an NT-proBNP level ≥900 pg/mL, irrespective of history of HF hospitalization. Key exclusion criteria included symptoms of hypotension or systolic blood pressure <95 mm Hg, estimated glomerular filtration rate <30 mL·min^–1^·1.73m^–^2 (or rapidly declining renal function), type 1 diabetes, and another condition likely to prevent patient participation in the trial or greatly limit life expectancy. A full list of exclusion criteria is provided in the design article.^13^

### Study Procedures

After the provision of informed consent, visit 1 started a 14-day screening period during which the trial inclusion and exclusion criteria were checked and baseline information was collected. Visit 2 was the randomization visit, and randomization was stratified based on diagnosis of type 2 diabetes (defined as an established diagnosis or a glycohemoglobin level of ≥6.5% [≥48 mmol/mol]) at screening. After randomization, follow-up visits took place at 14 and 60 days, and then at 120, 240, and 360 days, and every 4 months thereafter. The visit early after randomization (14 days) was included to check renal function and blood pressure (and for symptoms of hypotension, as well); this visit also allowed for adjustment of background diuretic or other nonessential therapies. Dose reduction to 5 mg of dapagliflozin or matching placebo (or discontinuation of study drug) was to be considered in case of an acute unexpected decline in the estimated glomerular filtration rate, volume depletion, or hypotension (or to avoid these conditions); however, dose uptitration (or reinitiation) was encouraged thereafter in all cases, where possible.

### Study Outcomes

The primary outcome was the composite of an episode of worsening HF or cardiovascular (CV) death, whichever occurred first. An episode of worsening HF was defined as either an unplanned hospitalization or an urgent visit resulting in intravenous therapy for HF. Secondary outcomes included the composite of the occurrence of HF hospitalization or CV death. Additional prespecified exploratory end points included a wider composite reflecting worsening HF, namely time to first occurrence of CV death, hospitalization for HF, an urgent HF visit, or worsening HF symptoms/signs leading to the initiation of new oral therapy or augmentation of existing oral treatment. Outpatient intensification of HF therapy was recorded by means of check box questions (yes/no) asked at each study visit and completed by the investigator on a specific case report form page: was there outpatient intensification of HF medication because of worsening symptoms/signs of HF, and, if so, was the dose of diuretic increased and sustained for at least 4 weeks, or was a new drug added for the treatment of worsening HF and sustained for at least 4 weeks? Further checkbox questions (yes/no) recorded any changes in the following medication classes: angiotensin-converting enzyme inhibitor, angiotensin receptor blocker, angiotensin receptor-neprilysin inhibitor, β-blocker, mineralocorticoid receptor antagonist, loop diuretic, other diuretics (eg, a thiazide), vasodilators, and other HF medications (eg, ivabradine, digitalis glycoside). We examined the effect of dapagliflozin, in comparison with placebo, on this expanded composite outcome and its components. Because reporting of episodes of outpatient worsening often occurred at the time of a study visit (or when another event occurred), it was not always certain whether such episodes occurred before or after a hospital admission, if both events occurred in a window between visits. Therefore, we performed a sensitivity analysis that included only episodes of outpatient worsening that were not preceded or followed by a HF hospitalization in the same time window between study visits.

We also examined the characteristics of patients experiencing each manifestation of nonfatal worsening of HF and CV death, and the subsequent survival of patients having a nonfatal episode of worsening. In the latter analysis, patients with an urgent HF visit or hospitalized for HF within a 30-day period after outpatient intensification of HF therapy were classified as either an urgent HF visit or HF hospitalization, respectively. If patients were hospitalized within 30 days after an urgent HF visit, they were classified as a HF hospitalization and not an urgent HF visit. The reference group consisted of patients who had none of these events during the trial (no-event group). In a sensitivity analysis, the time period used to determine independent events was shortened to a 7-day interval between events instead of 30 days. A further sensitivity analysis was performed limiting the definition of outpatient HF-worsening events to include only those in whom the dose of diuretics was increased and sustained for at least 4 weeks.

### Statistical Analysis

Baseline characteristics were compared between groups by using the Kruskal-Wallis test or analysis of variance (ANOVA) for continuous variables and the χ^2^ test for categorical variables. The effect of dapagliflozin in comparison with placebo on each outcome was examined by means of hazard ratio (HR) and 95% CIs derived from Cox proportional hazards models, stratified according to diabetes status, and adjusted for a history of hospitalization for HF and treatment-group assignment. The effect of dapagliflozin in comparison with placebo on the total (including recurrent) number of outpatient intensification of HF therapy events was examined by means of a semiparametric proportional rates model.^17^ The relative hazard of death after a first event was examined in a Cox proportional hazards model where an indicator of a patient’s first event type was entered in the model as a time-updated covariate (with follow-up time starting at randomization). The model was repeated with stratification by diabetes status and adjusted for treatment-group assignment and a history of hospitalization for HF. Further adjustment was performed for the following variables at baseline: age, sex, region, race, NYHA functional classification, left ventricular ejection fraction, body mass index, pulse, systolic blood pressure, serum creatinine, log NT-proBNP, history of atrial fibrillation, stroke, myocardial infarction, hypertension, ischemic cause, and the use of an implantable cardioverter defibrillator or cardiac resynchronization therapy or both. All *P* values are 2-sided and *P*<0.05 was considered significant. All analyses were performed using Stata version 16.0 (Stata Corp).

## Results

Of the 4744 patients randomly assigned in DAPA-HF, 604 (12.7%) had outpatient worsening of HF resulting in intensification of therapy, 33 (0.7%) had an urgent HF visit resulting in intravenous therapy, 549 (11.6%) were hospitalized for worsening HF, and 500 (10.5%) died of CV causes at some point during follow-up.

### First Episode of Worsening

The first nonfatal manifestation of HF was an outpatient episode resulting in intensification of HF therapy in 407 (8.6%) patients (without a subsequent urgent HF visit or hospitalization for HF within 30 days). An urgent HF visit resulting in intravenous therapy was the first manifestation in 20 (0.4%) patients (with no previous intensification of therapy or subsequent hospitalization for HF within 30 days). Hospitalization for HF, without a preceding outpatient episode of worsening or urgent HF visit, was the first manifestation in 489 (10.3%) patients. CV death was the first manifestation of worsening HF in 295 (6.2%) of randomly assigned patients.

The number of patients experiencing this expanded 4-component composite outcome (n=1211 patients) was substantially larger (38% more patients) than the number who experienced the conventional composite outcome of HF hospitalization or CV death (n=877). CV death was the first event in 295 of the patients, contributing to the expanded composite outcome (24% of first events). This was compared with 329 CV deaths as the first event among the 877 patients experiencing the narrower, 2-component, conventional composite outcome (38% of first events were a CV death).

### Baseline Characteristics

The Table shows the baseline characteristics of patients according to their first manifestation of HF worsening (and these characteristics for patients with no event). In comparison with patients with no worsening during follow-up, patients experiencing any worsening were in a more advanced NYHA functional class, had a much higher NT-proBNP level, and a higher heart rate, but lower systolic blood pressure and left ventricular ejection fraction. Patients with any form of worsening HF event reported a lower (worse) Kansas City Cardiomyopathy Questionnaire Total Symptom Score at baseline in comparison with those who had no worsening event. Patients with worsening HF also had a higher prevalence of diabetes, atrial fibrillation, and chronic kidney disease, and were more often treated with a diuretic and digoxin than those with no worsening.

**Table.**
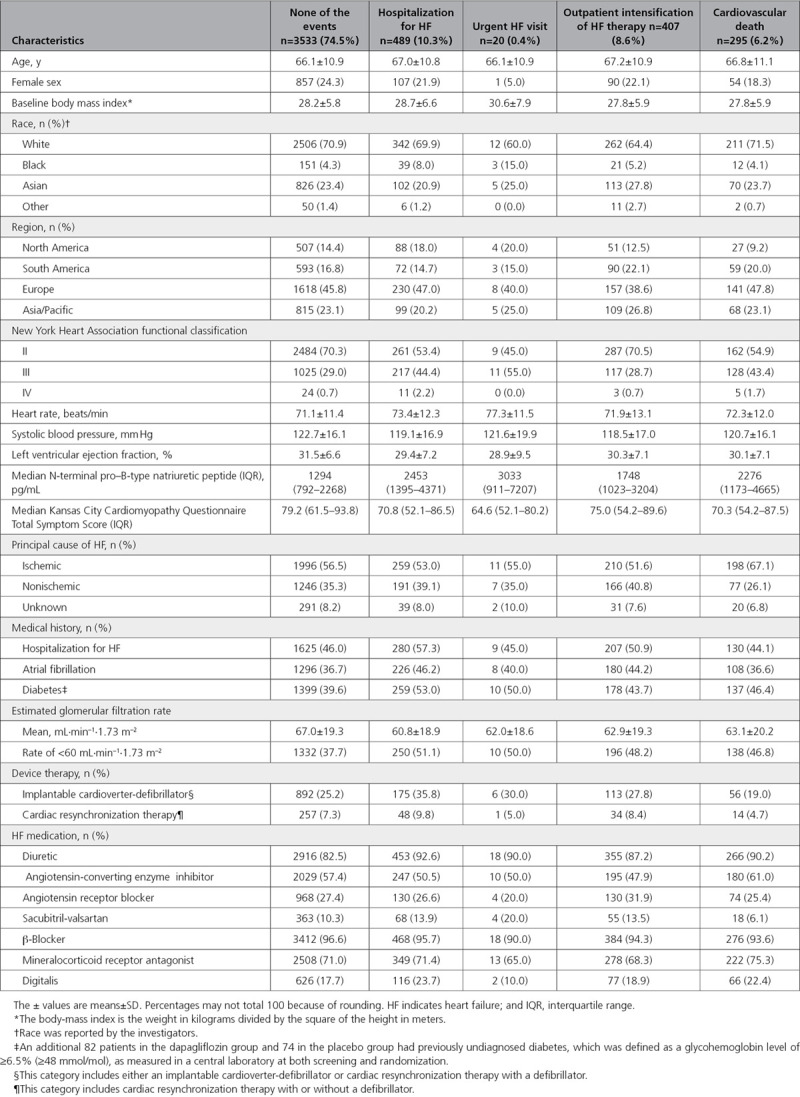
Baseline Characteristics of Patients With Different First Manifestations of HF Worsening, or None, or Experiencing Cardiovascular Death

In general, these trends were consistent for patients with each different nonfatal manifestation of worsening, but not always as clear for patients dying of a CV cause. Conversely, among patients dying of a CV cause, an ischemic cause was more common than among other patients. When considering outpatient worsening HF events in comparison with those who had no worsening event during follow-up, differences in NYHA functional class, heart rate, and the number of patients taking diuretics and digoxin were less apparent than the other manifestations of worsening HF.

### Treatments Given for Episodes of Outpatient Worsening

Of the 407 patients with outpatient worsening, 313 (76.9%) had intensification of diuretic treatment and 186 (45.7%) were started on additional HF therapy (these changes in treatment were not mutually exclusive). The addition of a new loop diuretic occurred in 34 of 407 (8.4%), and another diuretic in 18 (4.4%) patients not taking those medications at baseline. Additional changes to HF therapy (either a change in existing medication or a new treatment started) included an angiotensin-converting enzyme inhibitor or angiotensin receptor blocker in 72 (17.7%) patients, sacubitril/valsartan in 29 (7.1%), a β-blocker in 47 (11.5%), a mineralocorticoid receptor in 58 (14.3%), a vasodilator in 11 (2.7%), and another HF medication in 49 (12.0%). Data were unavailable for 18 of the 186 (9.7%) patients who had new HF therapy added.

### Effect of Dapagliflozin in Comparison With Placebo on Manifestations of Worsening HF

**Figure 1.**
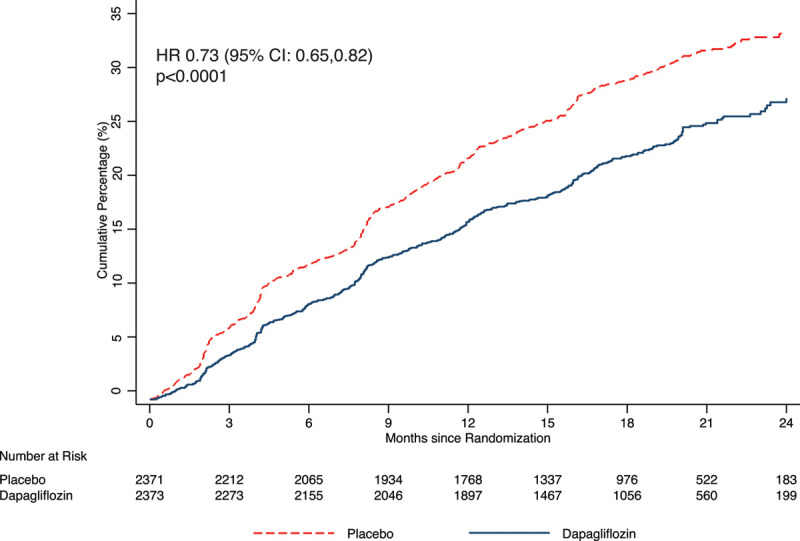
**Kaplan-Meier curves for the expanded composite outcome of time to first cardiovascular death, hospitalization for heart failure, urgent heart failure visit, or outpatient intensification of heart failure therapy, according to treatment group.** Hazard ratios (HR) and 95% CIs were estimated with the use of Cox regression models, stratified according to diabetes status, with a history of hospitalization for heart failure and treatment-group assignment as explanatory variables.

**Figure 2.**
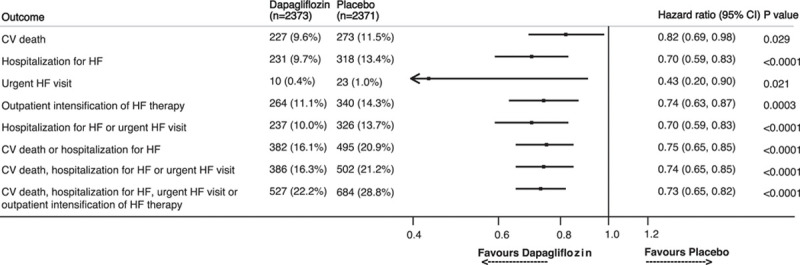
**Effect of dapagliflozin versus placebo on manifestations of worsening heart failure.** Hazard ratios and 95% CIs were estimated with the use of Cox regression models, stratified according to diabetes status, with a history of hospitalization for heart failure and treatment-group assignment as explanatory variables. CV indicates cardiovascular; and HF, heart failure.

In a prespecified expanded, 4-component, composite outcome of time to first HF hospitalization, urgent HF visit, outpatient worsening event, or CV death (total number of events=1211), there was a highly significant benefit of dapagliflozin over placebo: hazard ratio (HR), 0.73 (95% CI, 0.65–0.82), *P*<0.0001 (Figure 1). If the expanded composite outcome had been used as the primary outcome and the trial terminated once 844 events had accrued, the benefit of dapagliflozin would have been similar: HR, 0.71 (95% CI, 0.62–0.81); *P*<0.0001. In a sensitivity analysis considering only outpatient intensification events occurring without an HF hospitalization occurring in the same time window between 2 study visits, the beneficial effect of dapagliflozin in comparison with placebo was not modified with a HR of 0.71 (95% CI, 0.60–0.85). Dapagliflozin was superior to placebo in reducing each nonfatal manifestation of HF: the risk of hospitalization was reduced by 30% (95% CI, 17%–41%); *P*<0.0001; an urgent HF visit was reduced by 57% (95% CI, 10%–80%); *P*=0.021; and outpatient worsening was reduced by 26% (95% CI, 13%–37%; *P*=0.0003; Figure 2). When considering first and recurrent episodes of outpatient worsening, dapagliflozin reduced the risk of these events by 30% in comparison with placebo (rate ratio, 0.70 [95% CI, 0.59–0.84]; *P*=0.0001).

When patients with outpatient worsening were evaluated in more detail, the intensification of a diuretic for >4 weeks occurred significantly less frequently in patients treated with dapagliflozin: 213 (9.0%) on dapagliflozin in comparison with 283 (11.9%) on placebo (HR, 0.72 [95% CI, 0.61–0.87]; *P*=0.0004). The addition of new HF therapy occurred in 130 (5.5%) patients in the dapagliflozin group and 180 (7.6%) patients in the placebo group (HR, 0.70 [95% CI, 0.56–0.88]; *P*=0.002).

### Absolute Risk Reduction With Dapagliflozin in Comparison With Placebo Expressed as a Number Needed to Treat

The number of patients needed to treat with dapagliflozin over the median 18.2 months of follow-up in DAPA-HF to prevent 1 patient from experiencing an episode of fatal or nonfatal worsening was: 21 for the composite of CV death or HF hospitalization, 21 for the primary (3-component) composite, and 16 for the expanded 4-component composite.

### Risk of Death After a Nonfatal Episode of Worsening

Among the 407 patients in whom the first manifestation of worsening was an outpatient episode associated with the intensification of oral therapy, 323 of 407 (79.4%) did not have any further nonfatal episodes of worsening (of any type) or die of a CV cause; this number was 309 of 407 (75.9%) when considering death from any cause. Among the 407 patients with an outpatient episode of worsening, 48 (11.8%) subsequently died of a CV cause (65 [16.0%] died of any cause).

**Figure 3.**
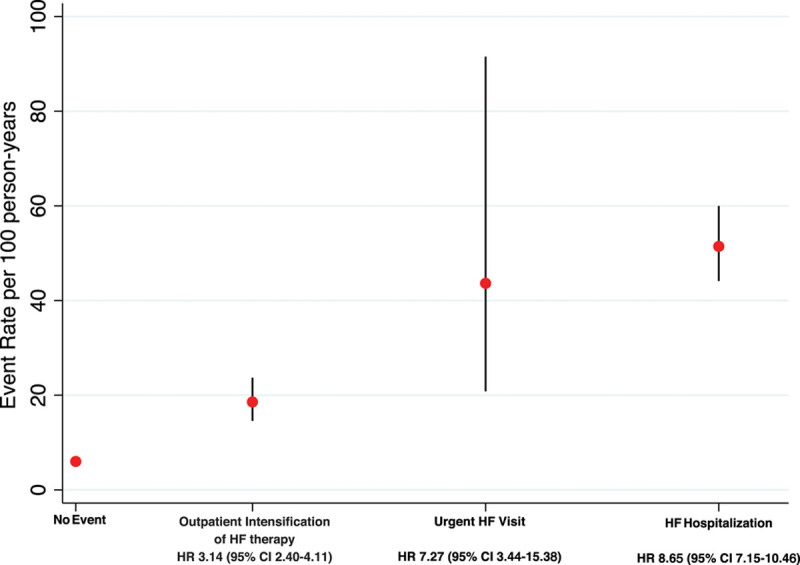
**Rate and risk of all-cause mortality after a first nonfatal heart failure worsening event.** The risk of each death from any cause relative to patient not experiencing an event after a first nonfatal heart failure worsening event is calculated by using Cox regression models with the event entered in the model as a time-updated covariate. HF indicates heart failure; and HR, hazard ratio.

Among the 20 patients in whom the first event was an urgent HF visit, 6 of 20 (30%) did not have any further nonfatal episodes of worsening (of any type) or die of a CV cause; this number was also 6 of 20 (30%) when considering death from any cause. Among the 20 patients in whom an urgent HF visit was the first nonfatal manifestation of worsening, 6 of 20 (30%) consequently died of a CV cause and 7 (35%) died of any cause. Of the 489 patients in whom the first event was a HF hospitalization, 204 (41.7%) did not have any further nonfatal episodes of worsening (of any type) or die of a CV cause; this number was 201 of 489 (41.1%) for death from any cause. Among the 489 patients in whom a HF hospitalization was the first manifestation of worsening HF, death from a CV cause or from any cause occurred in 151 (31%) and 163 (33%) patients, respectively,

Figure 3 shows the rate of death from any cause in patients with a first nonfatal manifestation of worsening HF, and the unadjusted risk of death by type of worsening, in comparison with patients with no worsening. The adjusted risk of death (in comparison with patients with no event) was ≈6-fold higher after a HF hospitalization (HR, 6.21 [95% CI, 5.07–7.62]) and ≈3-fold higher after an urgent HF visit (HR, 3.00 [95% CI, 1.39–6.48]) or after an outpatient episode of worsening (HR, 2.67 [95% CI, 2.03–3.52]; Table I in the Data Supplement). Similar results were found when the definition of outpatient episodes of worsening HF was limited to include only those in whom the dose of diuretic was increased and sustained for at least 4 weeks (Table II in the Data Supplement). The use of a 7-day interval between events to identify independent events rather than 30 days also did not alter the results (Table III in the Data Supplement).

## Discussion

In the present report, we provide new data, extending the evidence that dapagliflozin is beneficial in patients with HFrEF.^15,18^ Our findings confirm that worsening of HF, leading to the augmentation of oral therapy in the outpatient setting, is common and such events are of prognostic importance.^8–12^ These episodes of outpatient worsening were reduced substantially and significantly by dapagliflozin. This finding is important for patient management.

Although our patients had predominantly mild symptoms and were very well treated with conventional therapy, 1 in 8 patients experienced worsening of their HF requiring augmentation of oral therapy over a median follow-up period of just 18.2 months. Although not as prognostically important as episodes of HF worsening leading to hospital admission, episodes of worsening leading to augmentation of oral therapy in the outpatient setting were still associated with a ≈3-fold higher risk of death over the remainder of the trial, in comparison with patients without worsening. Adding these events to create an expanded time-to-first composite outcome illustrates the considerable residual rate of fatal or nonfatal worsening still experienced by patients with HFrEF despite excellent contemporary therapy. Specifically, in the placebo group, the rate of CV death or HF hospitalization (the conventional primary outcome in HF trials) was 15.3 per 100 person-years, but the event rate rose to 22.6 per 100 person-years for the broadest composite outcome (4-component composite), clearly reinforcing the continuing need for additional effective treatments in HFrEF.^11,12^ Thus, focusing only on HF hospitalization underestimates the frequency of clinical worsening in HFrEF, overall, and fails to recognize the other manifestations of worsening that have serious implications, as we have highlighted previously.^11^ These episodes of outpatient worsening may be all-the-more important given the current emphasis on attempting to minimize hospital admissions in patients with HF.^5,6^

The rate of outpatient HF worsening resulting in augmentation of oral therapy in DAPA-HF was higher than the rate observed in the PARADIGM-HF trial (Prospective Comparison of ARNI with ACEI to Determine Impact on Global Mortality and Morbidity in Heart Failure), in keeping with the higher rates of HF hospitalization and CV death in DAPA-HF.^11^ The rate of orally treated episodes of worsening was much higher than outpatient worsening leading to intravenous treatment, a finding consistent with PARADIGM-HF, other trials, and clinical experience.^8–12^

Therefore, the 2 most common nonfatal manifestations of worsening HF captured in this analysis clearly were outpatient episodes leading to the augmentation of oral therapy and hospital admission. Treatment with dapagliflozin decreased each of these. Dapagliflozin reduced the risk of outpatient worsening by 26% (HR, 0.74 [95% CI, 0.63–0.87]); the magnitude of this relative risk reduction was consistent with that observed for HF hospitalization (HR, 0.70 [95% CI, 0.59–0.83]). This new finding reinforces the important incremental therapeutic benefit of dapagliflozin in HF.^15,19^ Both the relative and absolute risk reductions of the expanded composite outcome with dapagliflozin, added to conventional therapy, were substantial. The number of patients needed to treat to prevent 1 episode of fatal or nonfatal worsening was only 16, over the median duration of 18.2 months. Although this small number reflects a large absolute benefit, that overall benefit consists of events of quite different clinical significance, ranging from death from CV causes to episodes of worsening symptoms/signs treated in the outpatient setting with oral therapy. If only more severe events (HF hospitalization or CV death) are included, the number of patients needed to treat rises to 21 and, for CV death alone, the number of patients needed to treat is 52. The prognostic importance of these episodes of outpatient worsening, coupled with the availability of therapeutic interventions that reduce them, and other adverse outcomes, argues for efforts to highlight the clinical significance of outpatient worsening to all practitioners involved in care of these patients, including internists, primary care physicians, and nurses.^11,12^

The finding in both DAPA-HF and PARADIGM-HF that outpatient worsening is much more likely to be treated with oral than intravenous treatment argues for revision of the uniform definitions for CV and stroke outcomes developed by the Standardized Data Collection for Cardiovascular Trials Initiative and the US Food and Drug Administration.^7^ These require use of intravenous therapy for confirmation of an episode of outpatient worsening. The similar prognostic importance of outpatient worsening, irrespective of whether therapy is given intravenously, and the response of both types of worsening to efficacious therapy support a change in this guidance.

As with other similar studies, there are some limitations. Most patients in DAPA-HF were in NYHA class II at the time of randomization and the duration of follow-up was relatively short. The rate of events may differ according to HF severity and the proportions of each type of event may differ according to HF severity and duration of follow-up. These limitations could affect the generalizability of our results. We did not collect information on the specific symptoms and signs investigators used to identify worsening of HF in the outpatient setting. Documentation of this information might be considered if outpatient episodes of worsening are incorporated into the primary outcome of a future trial, and such episodes might also be adjudicated by an end point committee. We also did not document visits to an emergency department that did not lead to hospital admission, although we know from PARADIGM-HF that these are infrequent, and some may have been identified as episodes of worsening leading to the use of intravenous therapy but not to hospitalization.^11^

In summary, in DAPA-HF, outpatient episodes of HF worsening were common, predictive of mortality, and reduced by dapagliflozin. A focus on HF hospitalization underestimates the frequency of HF worsening and ignores other events of prognostic importance that are preventable.

## Sources of Funding

The DAPA-HF trial (Dapagliflozin and Prevention of Adverse Outcomes in Heart Failure) was funded by AstraZeneca. Dr McMurray is supported by a British Heart Foundation Center of Research Excellence Grant RE/18/6/34217.

## Disclosures

Dr Docherty reports receiving grant support from Novartis and lecture fees from Eli Lilly. Dr Jhund reports receiving consulting fees, advisory board fees, and lecture fees from Novartis, advisory board fees from Cytokinetics, and grant support from Boehringer Ingelheim. Dr Anand reports receiving fees for serving as US national leader of a trial from AstraZeneca, fees for serving on a steering committee from ARCA Biopharma, Amgen, LivaNova, and Novartis, fees for serving on an end point committee from Boehringer Ingelheim, fees for serving as chair of a data and safety monitoring board from Boston Scientific, and advisory board fees from Zensun. Drs Bengtsson, Johanson, Lindholm, and Sjöstrand report being employed by AstraZeneca. Dr Böhm reports receiving lecture fees from Amgen, Bayer, Servier, Medtronic, Boehringer Ingelheim, Vifor Pharma, and Bristol-Myers Squibb, grant support and lecture fees from AstraZeneca, and grant support from Deutsche Forschungsgemeinschaft. Dr de Boer reports receiving grant support (paid to University Medical Center Groningen [UMCG]), consulting fees, and lecture fees from AstraZeneca, grant support (paid to UMCG) from Bristol-Myers Squibb, grant support (paid to UMCG) and consulting fees from Abbott, grant support (paid to UMCG) and lecture fees from Roche, and consulting fees from MandalMed and being a minority shareholder in scPharmaceuticals. Dr DeMets reports receiving consulting fees from Frontier Science, Actelion, Bristol-Myers Squibb, Medtronic, Boston Scientific, GlaxoSmithKline, and Merck, and consulting fees and being owner of DL DeMets Consulting. Dr Desai reports receiving consulting fees from Abbott, Biofourmis, Boston Scientific, Boehringer Ingelheim, DalCor Pharmaceuticals, and Regeneron, grant support (paid to Brigham and Women’s Hospital) and consulting fees from Alnylam Pharmaceuticals and Novartis, and advisory board fees from Corvidia and Relypsa. Dr Drozdz reports receiving personal fees from Berlin Chemie Menarini. Dr Howlett reports receiving grant support, consulting fees, and lecture fees from AstraZeneca, Boehringer Ingelheim, Novartis, and Servier, consulting fees and lecture fees from Novo Nordisk, consulting fees from Janssen, and grant support, consulting fees, lecture fees, and provision of drugs from Pfizer. Dr Inzucchi reports receiving advisory fees from AstraZeneca and Zafgen, lecture fees, consulting fees, fees for serving as a clinical trial publications committee member, reimbursement for medical writing, and travel support from Boehringer Ingelheim, fees for serving on a steering committee and travel support from Sanofi-Lexicon, lecture fees, consulting fees, and travel support from Merck, and advisory fees and travel support from vTv Therapeutics and Abbott-Alere. Dr Katova reports receiving fees for serving as national coordinator of a trial from Novartis and AstraZeneca. Dr Køber reports receiving lecture fees from Novartis and Bristol-Myers Squibb. Dr Kosiborod reports receiving grant support, honoraria, and research support from AstraZeneca, grant support and honoraria from Boehringer Ingelheim, and honoraria from Sanofi, Amgen, Novo Nordisk, Merck (Diabetes), Eisai, Janssen, Bayer, GlaxoSmithKline, Glytec, Intarcia Therapeutics, Novartis, Applied Therapeutics, Amarin, and Eli Lilly. Dr Langkilde reports being employed by and holding shares in AstraZeneca. Dr Martinez reports receiving personal fees from AstraZeneca as honoraria. Dr Merkely reports receiving personal fees from AstraZeneca and Servier. Dr Nicolaui reports receiving grants from AstraZeneca during the conduct of the study and personal fees from Amgen, Daiichi-Sankyo, and Servier, grants from AstraZeneca, Bristol-Meyers-Squibb, CLS Behring, Dalcor, Jansen, Novo Nordisk, and Vifor, and grants and personal fees from Bayer, Novartis, and Sanofi. Dr O’Meara reports receiving fees for serving on a clinical trial (paid to her institution), consulting fees, and lecture fees from AstraZeneca, Bayer, Amgen, and Novartis, consulting fees from Merck, fees for serving on a clinical trial (paid to her institution) from American Regent, and consulting fees and lecture fees from Pfizer and Boehringer Ingelheim. Dr Ponikowski reports receiving personal fees and fees to his institution from participation as an investigator in clinical trials from AstraZeneca during the conduct of the study and from Boehringer Ingelheim, Servier, Novartis, Berlin-Chemie, Bayer, Renal Guard Solutions, Pfizer, Respicardia, Cardiorentis, and Cibiem; grants, personal fees, and fees to his institution from Impulse Dynamics; and fees to his institution from Vifor, Corvia, and Revamp Medical. Dr Sabatine reports receiving grant support (paid to Brigham and Women’s Hospital) and consulting fees from Amgen, AstraZeneca, Intarcia Therapeutics, Janssen Research and Development, the Medicines Company, MedImmune, Merck, and Novartis, receiving consulting fees from Anthos Therapeutics, Bristol-Myers Squibb, CVS Caremark, DalCor Pharmaceuticals, Dyrnamix, Esperion, IFM Therapeutics, and Ionis Pharmaceuticals, receiving grant support (paid to Brigham and Woman’s Hospital) from Bayer, Daiichi Sankyo, Eisai, GlaxoSmithKline, Pfizer, Poxel, Quark Pharmaceuticals, and Takeda Pharmaceutical, and serving as a member of the TIMI Study Group, which receives grant support (paid to Brigham and Women’s Hospital) from Abbott, Aralez Pharmaceuticals, Roche, and Zora Biosciences. Dr Solomon reports receiving grant support and consulting fees (all fees listed paid to Brigham and Women’s Hospital) from Alnylam Pharmaceuticals, Amgen, AstraZeneca, Bristol-Myers Squibb, Gilead Sciences, GlaxoSmithKline, MyoKardia, Novartis, Theracos, Bayer, and Cytokinetics, grant support from Bellerophon Therapeutics, Celladon, Ionis Pharmaceuticals, Lonestar Heart, Mesoblast, Sanofi Pasteur, and Eidos Therapeutics, consulting fees from Akros Pharma, Corvia Medical, Ironwood Pharma, Merck, Roche, Takeda Pharmaceutical, Quantum Genomics, AOBiome, Cardiac Dimensions, Tenaya Therapeutics, and Daiichi Sankyo, and fees for serving on a data and safety monitoring board from Janssen. Dr Tereshchenko reports receiving lecture fees from Servier, Pfizer, Novartis, and Boehringer Ingelheim. Dr Verma reports receiving grant support, lecture fees, and advisory board fees from AstraZeneca, Boehringer Ingelheim, Bayer, Janssen, and Merck, lecture fees from Sun Pharmaceutical Industries and EOCI Pharmacomm, grant support and advisory board fees from Amgen, and lecture fees and advisory board fees from Sanofi and Eli Lilly. Dr. McMurray reports receiving fees (all fees listed paid to Glasgow University) for serving on a steering committee from Bayer, DalCor Pharmaceuticals, and Bristol-Myers Squibb; fees for serving on a steering committee, fees for serving on an end point committee, and travel support from Cardiorentis; fees for serving on a steering committee and travel support from Amgen, Oxford University-Bayer, AbbVie; fees for serving as principal investigator of a trial and travel support from Theracos; fees for serving on a data and safety monitoring committee from Pfizer and Merck; fees for serving on an executive committee, fees for serving as coprincipal investigator of a trial, fees for serving on a steering committee, travel support, and advisory board fees from Novartis; fees for serving as coprincipal investigator for a trial, fees for serving on a steering committee, and travel support from GlaxoSmithKline; and fees for serving on a steering committee, fees for serving on an end point adjudication committee and travel support from Vifor Pharma-Fresenius. The other authors report no conflicts.

## Supplemental Materials

Data Supplement Tables I–III

## Supplementary Material



## References

[1] Swedberg K Komajda M Böhm M Borer JS Ford I Dubost-Brama A Lerebours G Tavazzi L; SHIFT Investigators Ivabradine and outcomes in chronic heart failure (SHIFT): a randomised placebo-controlled study. Lancet. 2010;376:875–885. doi: 10.1016/S0140-6736(10)61198-1 20801495 10.1016/S0140-6736(10)61259-7

[2] Zannad F McMurray JJ Krum H van Veldhuisen DJ Swedberg K Shi H Vincent J Pocock SJ Pitt B; EMPHASIS-HF Study Group Eplerenone in patients with systolic heart failure and mild symptoms. N Engl J Med. 2011;364:11–21. doi: 10.1056/NEJMoa100949221073363

[3] McMurray JJ Packer M Desai AS Gong J Lefkowitz MP Rizkala AR Rouleau JL Shi VC Solomon SD Swedberg K; PARADIGM-HF Investigators and Committees Angiotensin-neprilysin inhibition versus enalapril in heart failure. N Engl J Med. 2014;371:993–1004. doi: 10.1056/NEJMoa1409077 25176015 10.1056/NEJMoa1409077

[4] McMurray JJ Krum H Abraham WT Dickstein K Køber LV Desai AS Solomon SD Greenlaw N Ali MA Chiang Y; ATMOSPHERE Committees Investigators Aliskiren, enalapril, or aliskiren and enalapril in heart failure. N Engl J Med. 2016;374:1521–1532. doi: 10.1056/NEJMoa1514859 27043774 10.1056/NEJMoa1514859

[5] Wasfy JH Zigler CM Choirat C Wang Y Dominici F Yeh RW Readmission rates after passage of the hospital readmissions reduction program: a pre-post analysis. Ann Intern Med. 2017;166:324–331. doi: 10.7326/M16-0185 28024302 10.7326/M16-0185 PMC5507076

[6] Vidic A Chibnall JT Hauptman PJ Heart failure is a major contributor to hospital readmission penalties. J Card Fail. 2015;21:134–137. doi: 10.1016/j.cardfail.2014.12.002 25498757 10.1016/j.cardfail.2014.12.002

[7] Hicks KA Mahaffey KW Mehran R Nissen SE Wiviott SD Dunn B Solomon SD Marler JR Teerlink JR Farb A; Standardized Data Collection for Cardiovascular Trials Initiative (SCTI) 2017 Cardiovascular and stroke endpoint definitions for clinical trials. J Am Coll Cardiol. 2018;71:1021–1034. doi: 10.1016/j.jacc.2017.12.048 29495982 10.1016/j.jacc.2017.12.048

[8] Skali H Dwyer EM Goldstein R Haigney M Krone R Kukin M Lichstein E McNitt S Moss AJ Pfeffer MA Prognosis and response to therapy of first inpatient and outpatient heart failure event in a heart failure clinical trial: MADIT-CRT. Eur J Heart Fail. 2014;16:560–565. doi: 10.1002/ejhf.71 24578164 10.1002/ejhf.71

[9] Curtis AB Worley SJ Adamson PB Chung ES Niazi I Sherfesee L Shinn T Sutton MS; Biventricular versus Right Ventricular Pacing in Heart Failure Patients with Atrioventricular Block (BLOCK HF) Trial Investigators Biventricular pacing for atrioventricular block and systolic dysfunction. N Engl J Med. 2013;368:1585–1593. doi: 10.1056/NEJMoa1210356 23614585 10.1056/NEJMoa1210356

[10] Bristow MR Saxon LA Boehmer J Krueger S Kass DA De Marco T Carson P DiCarlo L DeMets D White BG; Comparison of Medical Therapy, Pacing, and Defibrillation in Heart Failure (COMPANION) Investigators Cardiac-resynchronization therapy with or without an implantable defibrillator in advanced chronic heart failure. N Engl J Med. 2004;350:2140–2150. doi: 10.1056/NEJMoa032423 15152059 10.1056/NEJMoa032423

[11] Okumura N Jhund PS Gong J Lefkowitz MP Rizkala AR Rouleau JL Shi VC Swedberg K Zile MR Solomon SD; PARADIGM-HF Investigators and Committees* Importance of clinical worsening of heart failure treated in the outpatient setting: evidence from the prospective comparison of ARNI with ACEI to determine impact on global mortality and morbidity in heart failure trial (PARADIGM-HF). Circulation. 2016;133:2254–2262. doi: 10.1161/CIRCULATIONAHA.115.020729 27143684 10.1161/CIRCULATIONAHA.115.020729

[12] Greene SJ Mentz RJ Felker GM Outpatient worsening heart failure as a target for therapy: a review. JAMA Cardiol. 2018;3:252–259. doi: 10.1001/jamacardio.2017.5250 29387880 10.1001/jamacardio.2017.5250 PMC7474527

[13] McMurray JJV DeMets DL Inzucchi SE Køber L Kosiborod MN Langkilde AM Martinez FA Bengtsson O Ponikowski P Sabatine MS; DAPA-HF Committees and Investigators A trial to evaluate the effect of the sodium-glucose co-transporter 2 inhibitor dapagliflozin on morbidity and mortality in patients with heart failure and reduced left ventricular ejection fraction (DAPA-HF). Eur J Heart Fail. 2019;21:665–675. doi: 10.1002/ejhf.1432 30895697 10.1002/ejhf.1432 PMC6607736

[14] McMurray JJV DeMets DL Inzucchi SE Køber L Kosiborod MN Langkilde AM Martinez FA Bengtsson O Ponikowski P Sabatine MS; DAPA-HF Committees and Investigators The Dapagliflozin And Prevention of Adverse-outcomes in Heart Failure (DAPA-HF) trial: baseline characteristics. Eur J Heart Fail. 2019;21:1402–1411. doi: 10.1002/ejhf.1548 31309699 10.1002/ejhf.1548

[15] McMurray JJV Solomon SD Inzucchi SE Køber L Kosiborod MN Martinez FA Ponikowski P Sabatine MS Anand IS Bělohlávek J; DAPA-HF Trial Committees and Investigators Dapagliflozin in patients with heart failure and reduced ejection fraction. N Engl J Med. 2019;381:1995–2008. doi: 10.1056/NEJMoa1911303 31535829 10.1056/NEJMoa1911303

[16] AstraZeneca AstraZeneca Clinical Trials - Disclosure Commitment.. https://astrazenecagrouptrials.pharmacm.com/ST/Submission/Disclosure.

[17] Lin DY Wei LJ Yang I Ying Z Semiparametric regression for the mean and rate functions of recurrent events. J R Stat Soc Series B Stat Methodol. 2000;62:711–730

[18] Petrie MC Verma S Docherty KF Inzucchi SE Anand I Belohlávek J Böhm M Chiang CE Chopra VK de Boer RA Effect of dapagliflozin on worsening heart failure and cardiovascular death in patients with heart failure with and without diabetes. JAMA. 2020;323:1353–1368. doi: 10.1001/jama.2020.1906 32219386 10.1001/jama.2020.1906 PMC7157181

[19] Docherty KF Jhund PS Inzucchi SE Køber L Kosiborod MN Martinez FA Ponikowski P DeMets DL Sabatine MS Bengtsson O Effects of dapagliflozin in DAPA-HF according to background heart failure therapy. Eur Heart J. 2020;41:2379–2392. doi: 10.1093/eurheartj/ehaa183 32221582 10.1093/eurheartj/ehaa183 PMC7327533

