# Supplementary material for: Effect of Dapagliflozin on Outpatient Worsening of Patients With Heart Failure and Reduced Ejection Fraction: A Prespecified Analysis of DAPA-HF
Source: Circulation. 2020 Sep 4;142(17):1623–32. doi: 10.1161/CIRCULATIONAHA.120.047480 (PMC7580857; doi:10.1161/CIRCULATIONAHA.120.047480)
Supplement: Supplementary file 1 [file cir-142-1623-s001.pdf]

Effect of dapagliflozin on outpatient worsening of patients with heart failure and reduced ejection fraction: A prespecified analysis of DAPA-HF.

SUPPLEMENTAL MATERIAL

Supplemental Table I: Risk of death from any cause after a hospitalization for heart failure, urgent heart failure visit, and intensification of therapy for heart failure using a cox model with event type as the first event experienced.

	No Event	Hospitalization for HF	Urgent HF Visit	Intensification of HF Therapy
**Each event as the first event experienced in a time-updated model, hazard ratio (95% CI)**				
Adjusted for randomized treatment and prior HF hospitalization	1	8.36 (6.89-10.15)	7.04 (3.32-14.90)	3.12 (2.38-4.09)
Adjusted for randomized treatment , prior HF hospitalization and baseline covariates*	1	6.21 (5.07-7.62)	3.00 (1.39-6.48)	2.67 (2.03-3.52)

All models stratified by diabetes status.

*Adjusted for randomized treatment, age, sex, region, race, NYHA functional classification, left ventricular ejection fraction, body mass index, pulse, systolic blood pressure, serum creatinine, log NT-proBNP, history of previous HF hospitalization, atrial fibrillation, stroke, myocardial infarction, hypertension , ischemic etiology and use of implantable cardioverter defibrillator and/or cardiac resynchronization therapy.

Supplemental Table II: Risk of death from any cause after a hospitalization for heart failure, urgent heart failure visit, and intensification of therapy for heart failure using a cox model with event type as the first event experienced – sensitivity analysis limiting the definition of outpatient HF worsening events to include only those in which the dose of diuretics was increased and sustained for at least four weeks.

	**No Event**	**Hospitalization for HF**	**Urgent HF Visit**	**Intensification of HF Therapy (diuretics only)**
**Each event as the first event experienced in a time-updated model, hazard ratio (95% CI)**				
Unadjusted	1	8.53 (7.06-10.30)	6.50 (3.07-13.75)	3.38 (2.54-4.49)
Adjusted for randomized treatment and prior HF hospitalization	1	8.26 (6.81-10.00)	6.30 (2.97-13.34)	3.37 (2.53-4.48)
Adjusted for randomized treatment , prior HF hospitalization and baseline covariates*	1	6.14 (5.01-7.51)	2.85 (1.32-6.14)	2.82 (2.11-3.77)

All adjusted models stratified by diabetes status.

*Adjusted for randomized treatment, age, sex, region, race, NYHA functional classification, left ventricular ejection fraction, body mass index, pulse, systolic blood pressure, serum creatinine, log NT-proBNP, history of previous HF hospitalization, atrial fibrillation, stroke, myocardial infarction, hypertension , ischemic etiology and use of implantable cardioverter defibrillator and/or cardiac resynchronization therapy.

Supplemental Table III: Risk of death from any cause after a hospitalization for heart failure, urgent heart failure visit, and intensification of therapy for heart failure using a cox model with event type as the first event experienced – sensitivity analysis using a 7-day period between events to identify independent events

	**No Event**	**Hospitalization for HF**	**Urgent HF Visit**	**Intensification of HF Therapy (diuretics only)**
**Each event as the first event experienced in a time-updated model, hazard ratio (95% CI)**				
Unadjusted	1	8.32 (6.86-10.09)	7.07 (3.34-14.96)	3.52 (2.72-4.54)
Adjusted for randomized treatment and prior HF hospitalization	1	8.03 (6.60-9.78)	6.84 (3.23-14.49)	3.50 (2.70-4.52)
Adjusted for randomized treatment , prior HF hospitalization and baseline covariates*	1	5.96 (4.85-7.33)	3.04 (1.41-6.59)	2.96 (2.28-3.84)

All adjusted models stratified by diabetes status.

*Adjusted for randomized treatment, age, sex, region, race, NYHA functional classification, left ventricular ejection fraction, body mass index, pulse, systolic blood pressure, serum creatinine, log NT-proBNP, history of previous HF hospitalization, atrial fibrillation, stroke, myocardial infarction, hypertension , ischemic etiology and use of implantable cardioverter defibrillator and/or cardiac resynchronization therapy.
